# Interchannel Interference and Mitigation in Distributed MIMO RF Sensing

**DOI:** 10.3390/s21227496

**Published:** 2021-11-11

**Authors:** Sahil Waqar, Matthias Pätzold

**Affiliations:** Faculty of Engineering and Science, University of Agder, 4630 Kristiansand, Norway; matthias.paetzold@uia.no

**Keywords:** interchannel interference, distributed MIMO, 3D channel model, sensor network, millimeter wave (mm-Wave), FMCW, micro-Doppler signatures, RF sensing

## Abstract

In this paper, we analyze and mitigate the cross-channel interference, which is found in multiple-input multiple-output (MIMO) radio frequency (RF) sensing systems. For a millimeter wave (mm-Wave) MIMO system, we present a geometrical three-dimensional (3D) channel model to simulate the time-variant (TV) trajectories of a moving scatterer. We collected RF data using a state-of-the-art radar known as Ancortek SDR-KIT 2400T2R4, which is a frequency-modulated continuous wave (FMCW) MIMO radar system operating in the K-band. The Ancortek radar is currently the only K-band MIMO commercial radar system that offers customized antenna configurations. It is shown that this radar system encounters the problem of interference between the various subchannels. We propose an optimal approach to mitigate the problem of cross-channel interference by inducing a propagation delay in one of the channels and apply range gating. The measurement results prove the effectiveness of the proposed approach by demonstrating a complete elimination of the interference problem. The application of the proposed solution on Ancortek’s SDR-KIT 2400T2R4 allows resolving all subchannel links in a distributed MIMO configuration. This allows using MIMO RF sensing techniques to track a moving scatterer (target) regardless of its direction of motion.

## 1. Introduction

The birth of radar in the first half of the last century led to numerous research studies and advances in the field. Although radar systems were originally developed for military surveillance tasks, modern radars have found many applications in our daily lives due to their continuous development over the decades. Conventionally, radar systems were limited to official or governmental entities, but now their smaller form factor, lower cost, higher precision, and easier handling have led to more general utilization. Conventional applications of radars have been aerial [1] and terrestrial [2] traffic control, missile and aerial defense [3], altimetry [4], naval surveillance [5], weather surveillance [6], and astronomy [7], whereas the contemporary radar systems have also been employed in modern medicine [8], autonomous vehicles [9,10,11], geology [12], building security, human activity recognition systems [13,14,15,16], and even in consumer electronics such as mobile phones [17] (serving as a gesture recognition system). It is now safe to assert that the idea of radar sensors being ubiquitous is not far-fetched anymore due to their miniaturization, affordability, and utility. For a non-trivial problem such as autonomous driving in automotive engineering, several types of radar systems (short-range, medium-range, and long-range) [18] are typically integrated to achieve the desired performance, especially under adverse lighting conditions, where other sensing modalities do not perform as required.

A radar system transmits electromagnetic waves and processes the received backscattered waves to estimate one or more parameters of an object present in the environment. Depending on the type of radar, it may measure the range, Doppler (or micro-Doppler) signature, and angular information of a target within certain limitations. Depending on the problem, a radar may be designed and deployed as a continuous wave (CW) radar [19], frequency-modulated continuous wave (FMCW) radar [20], pulsed radar [21], bistatic radar, monopulse radar [22,23], synthetic aperture radar (SAR) [24], digital beamforming (DBF) multiple-input multiple-output (MIMO) radar in a monostatic configuration [25,26], or distributed MIMO radar [27,28,29]. Recently, short- to medium-range FMCW radars have been gaining increasing attention for commercial indoor and outdoor applications. For instance, the authors of [30,31] have used a K-band FMCW radar system in indoor settings to monitor human vital functions. More recently, FMCW radar systems operating in the W-band have been adopted for more sophisticated applications, such as sign language recognition [32], multimodal traffic monitoring [33], and skeletal posture estimation [34].

Generally, radar systems suppress the static clutter by filtering out the zero-Doppler frequency components from the received signal, which prevents detection and tracking of the scatterer’s motion perpendicular to its boresight. Thus, to acquire the scatterer’s motion information from multiple aspect angles, the deployment of a single-input single-output (SISO) radar or a monostatic MIMO radar is not a suitable choice. Instead, with the idea of macrodiversity, a distributed MIMO radar system or a multistatic radar network is preferred to circumvent the shortcomings of the aforementioned radar configurations. It is in this context that we will focus our attention on the deployment of a distributed MIMO radar system in indoor environments. For different application areas, researchers are investigating different target–antenna configurations while leaning towards multistatic radar networks. For example, the authors of [35] deployed a network known as NetRAD for the detection of armed/unarmed personnel, and the authors of [36] report the use of a commercial DWM1000 ultra wideband wireless transceiver module in a multistatic configuration to track a moving person in a cluttered indoor or outdoor environment.

The probability of mutual interference between radar systems is increasing gradually as commercial radars become more widely used. In distributed MIMO radar systems, cross-channel interference exists between the different nodes of a multistatic radar network. For this research, we chose a radar system that uses the time division multiple access (TDMA) scheme to avoid cross-channel interference. In the TDMA mode, the transmitters of a MIMO radar system operate in different time slots. As part of the physical channel characteristics, it is also imperative for the system performance to consider the interchannel radio frequency (RF) isolation inside the RF circuitry. In case of RF leakage in MIMO radar subchannels, the received signals are of the same order of magnitude for all receiver channels. For a consumer grade hardware that undergoes such RF leakage, the signal from one receiver leaks into the other receiver, and vice versa, making it impossible to separate the subchannels from each other. The problem is then to distinguish the received signal once impaired by RF-leakage from the co-channel signals. The interference problems arising due to the RF leakage between the RF chains cannot be resolved by the TDMA scheme, because the TDMA scheme is only effective against cross-channel interference if good RF-isolation is ensured beforehand. Thus, for such consumer grade MIMO radar systems, we propose a robust approach in this paper to solve the interference problem.

To estimate the trajectories of a non-stationary scatterer from different aspect angles in a cluttered indoor environment, we adopt Ancortek’s commercial MIMO radar system SDR-KIT 2400T2R4, which operates in the 24–26 GHz frequency band. It has in aggregate six independent physical RF chains: two transmitter chains and four receiver chains. For this research, we utilize Ancortek’s 2×4 MIMO radar system in a 2×2 configuration for simplicity. Ancortek’s radar system is currently the only commercially available MIMO radar system that offers the flexibility to distribute its antennas and to process all eight MIMO subchannel links individually. We distribute two pairs of collocated transmitter–receiver antennas in an indoor setting to illuminate a non-stationary scatterer from different aspect angles. The problem of cross-channel interference arises in Ancortek’s MIMO radar system even with the utilization of the TDMA scheme. Furthermore, we will point out that Ancortek’s SDR-KIT 2400T2R4 has a very poor interchannel RF isolation, which leads to incorrect measurements of the mean Doppler shift. Thus, without any hardware or firmware alteration, there is no known optimal solution to effectively isolate the different RF MIMO channel links. The problem of interference in the Ancortek radar has also been reported by the authors of [37], where they have subtracted the spectrograms to alleviate the interference problem. The solution proposed in [37] is suboptimal and non-robust; it works when the interference component is smaller than the subchannel’s main component and fails when the interference component is of the order of the magnitude of the main component of the subchannel. Therefore, in this paper, we propose an optimal and robust solution that completely eradicates the problem of cross-channel interference. The proposed solution performs effectively even when the interference component is stronger than the subchannel’s main component. Although our focus is on Ancortek’s radar, similar interference problems may also persist for future commercially available MIMO radar sensors. Thus, for such MIMO radar systems, the proposed solution can be adopted without entailing any hardware or firmware modifications. Additionally, the proposed solution also helps alleviate the maximum measurable velocity or the pulse repetition frequency (PRF) of the radar by completely avoiding the TDMA scheme, and still being able to segregate the MIMO channel links.

The principal contributions of this paper are as follows:

1. For a MIMO radar system whose antennas are distributed in an indoor cluttered environment, we present a system-theoretical approach to simulate the time-varying (TV) trajectories of a scatterer with arbitrary antenna placements.
2. We illuminate a non-stationary scatterer from different aspect angles (by deploying two pairs of collocated transmitter-receiver antennas) to analyze the TV micro-Doppler spectrogram, TV radial velocity profile, and TV mean Doppler shift.
3. For Ancortek’s SDR-KIT 2400T2R4 distributed MIMO radar system, we highlight the problem of cross-channel interference. We propose an optimal and robust solution to completely eradicate the interference components without modifying the hardware or firmware of the MIMO radar system.
4. We conduct experiments to verify the effectiveness of the proposed solution by successfully segregating the measured MIMO subchannels’ data.
5. We cross-validate the analytical model and the proposed solution of the interference problem by comparing the simulation results with the measurement results.

The organization of the paper is as follows. Section 2 formulates the interference problems that persist in Ancortek’s SDR-KIT 2400T2R4 distributed MIMO radar system. The geometrical 3D indoor channel model and the radar system model are presented in Section 3 and Section 4, respectively. Section 5 elucidates the proposed solution to the interference problem. The simulation results and the measurement results are discussed in Section 6. Finally, Section 7 summarizes our results and draws the conclusions.

## 2. Problem Description

**Figure 1:**
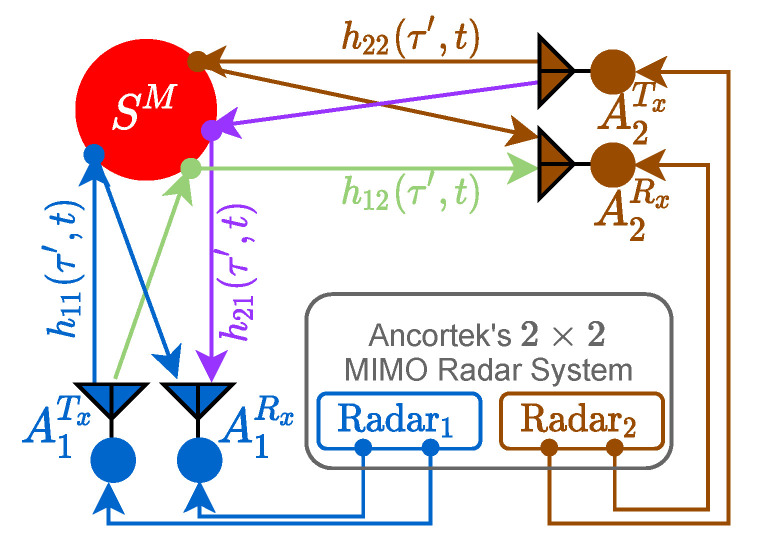
Two radar subsystems forming a 2×2 MIMO radar system in the presence of a single moving scatterer $S^{M}$.

Capturing and tracking nonlinear trajectories of moving scatterers indoors by means of RF-sensing modalities presents a number of challenges. One major challenge is to detect the scatterer trajectories regardless of the radar’s aspect angle, which requires multiple RF sensors. Therefore, for our experiments, a software-defined radar (SDR) known as Ancortek SDR-KIT 2400T2R4 has been configured in a 2×2 MIMO radar setup in the presence of a single moving scatterer $S^{M}$ as illustrated in Figure 1. The 2×2 MIMO radar system is composed of two radar subsystems, denoted as ${\mathrm{Radar}}_{1}$ and ${\mathrm{Radar}}_{2}$. The first subsystem (${\mathrm{Radar}}_{1}$) is equipped with the transmitter antenna $A_{1}^{T_{x}}$ and the receiver antenna $A_{1}^{R_{x}}$, whereas the second subsystem (${\mathrm{Radar}}_{2}$) is composed of the transmitter antenna $A_{2}^{T_{x}}$ and the receiver antenna $A_{2}^{R_{x}}$. Although the two radar subsystems are part of the same Ancortek system, they have identical but completely separate signal processing units.

The wireless channel link from the transmitter antenna $A_{i}^{T_{x}}$ to the receiver antenna $A_{j}^{R_{x}}$ via the scatterer $S^{M}$ is denoted by $A_{i}^{T_{x}}$–$A_{j}^{R_{x}}$, where i,j∈{1,2}. The time-variant channel impulse response (TV-CIR) $h_{ij}\left(\tau^{\prime },t\right)$ corresponds to the link $A_{i}^{T_{x}}$–$A_{j}^{R_{x}}$ as illustrated in Figure 1. Moreover, the two subradars operate in the same frequency range but in different time slots. Each subradar is assigned a different time slot according to the TDMA scheme to avoid cross-channel interference between the two subradars. In TDMA mode, the TV-CIRs $h_{21}\left(\tau^{\prime },t\right)$ and $h_{12}\left(\tau^{\prime },t\right)$ do not interfere with $h_{11}\left(\tau^{\prime },t\right)$ and $h_{22}\left(\tau^{\prime },t\right)$, respectively, but this is not true for the Ancortek SDR-KIT 2400T2R4 MIMO radar. The commercially available Ancortek MIMO radar system poses the problem of cross-channel interference even in TDMA mode due to its poor interchannel RF isolation. It is vital for system designers to ensure a good RF-isolation in the MIMO radar RF-circuitry, but such insurance is hard to realize for miniaturized and cost-effective RF circuits. Here, this phenomenon of RF leakage between the physical RF channels has been first investigated for the Ancortek radar because it is currently the only commercially available K-band radar that allows to distribute its antennas. However, the same problem may persist in future commercial MIMO radar systems. Note that this analysis provides guidelines for radar system designers to avoid cross-channel interference in their future designs. In addition, the analysis provides a performance criterion for the test and evaluation of the future FMCW MIMO radar systems. Note that the frequency division multiple access (FDMA) scheme is generally not preferred in commercial FMCW MIMO radar systems because of the associated complexity and cost. The FDMA approach limits the instantaneous bandwidth of an FMCW radar, which in turn limits the range resolution of the radar (see Section 4).

The TV-CIRs $h_{11}\left(\tau^{\prime },t\right)$ and $h_{22}\left(\tau^{\prime },t\right)$ are related to ${\mathrm{Radar}}_{1}$ and ${\mathrm{Radar}}_{2}$, respectively. Under ideal circumstances, ${\mathrm{Radar}}_{1}$ would only receive the signal corresponding to the wireless channel link $A_{1}^{T_{x}}$–$A_{1}^{R_{x}}$, and ${\mathrm{Radar}}_{2}$ would only receive the signal corresponding to the link $A_{2}^{T_{x}}$–$A_{2}^{R_{x}}$. However, due to the poor interchannel RF isolation of the Ancortek radar system, the receivers of the two radars strongly interfere with each other. This problem is independent of the channel impulse response length. The system is paused between switching from ${\mathrm{Radar}}_{1}$ to ${\mathrm{Radar}}_{2}$, but the two subsystems, i.e., ${\mathrm{Radar}}_{1}$ and ${\mathrm{Radar}}_{2}$, are part of one and the same MIMO radar system having a single RF printed circuit board (PCB). This RF circuit has poor RF isolation, due to which we encounter the problems of RF-leakage and cross-channel interference. The actual measured TV-CIRs ${\tilde{h}}_{11}\left(\tau^{\prime },t\right)$ and ${\tilde{h}}_{22}\left(\tau^{\prime },t\right)$ incorporating the problem of cross-channel interference are

(1) $${\tilde{h}}_{11}\left(\tau^{\prime },t\right)=h_{11}\left(\tau^{\prime },t\right)+\alpha_{22}^{11}h_{22}\left(\tau^{\prime },t\right)+\alpha_{12}^{11}h_{12}\left(\tau^{\prime },t\right)+\alpha_{21}^{11}h_{21}\left(\tau^{\prime },t\right)$$

and

(2) $${\tilde{h}}_{22}\left(\tau^{\prime },t\right)=h_{22}\left(\tau^{\prime },t\right)+\alpha_{11}^{22}h_{11}\left(\tau^{\prime },t\right)+\alpha_{12}^{22}h_{12}\left(\tau^{\prime },t\right)+\alpha_{21}^{22}h_{21}\left(\tau^{\prime },t\right)$$

respectively, where $\alpha_{ij}^{kk}$ is the weight corresponding to the TV-CIR of the interfering link for i,j,k∈{1,2}. The system model described by (1) takes into account that the measured TV-CIR ${\tilde{h}}_{11}\left(\tau^{\prime },t\right)$ comprises the desired component $h_{11}\left(\tau^{\prime },t\right)$ and the three undesired cross-channel interference components $\alpha_{22}^{11}h_{22}\left(\tau^{\prime },t\right)$, $\alpha_{12}^{11}h_{12}\left(\tau^{\prime },t\right)$, and $\alpha_{21}^{11}h_{21}\left(\tau^{\prime },t\right)$. Equation (2) presents an analogous system model for the cross-channel inference impairing the actual measured TV-CIR ${\tilde{h}}_{22}\left(\tau^{\prime },t\right)$. The weights $\alpha_{ij}^{kk}$ depend on the RF isolation between the subchannels of the MIMO radar system. An ideal MIMO radar system fulfills the condition $\alpha_{ij}^{kk}=0$, implying that ${\tilde{h}}_{ii}\left(\tau^{\prime },t\right)=h_{ii}\left(\tau^{\prime },t\right)$, but in practice, we have $\alpha_{ij}^{kk}\neq 0\;\forall \;i,j,k\in \left\{1,2\right\}$.

To demonstrate the practical relevance of the described problem, we study the cross-channel interference of the Ancortek MIMO radar. Therefore, we measure the nonlinear trajectories of a swinging pendulum in a 2×2 MIMO radar setup. Let us consider a swinging pendulum as a physical model for a moving scatterer $S^{M}$ as shown in Figure 1. The choice of a pendulum as a moving scatterer $S^{M}$ is appropriate as the trajectory of $S^{M}$ can be described by a mathematical reference model as shown in Section 6, which is important for the cross-validation of the experimental results. The two subradars are positioned on the two-dimensional orthogonal axes (x,y). This arrangement of subradars enables the overall system to capture the scatterer’s motion in the horizontal plane, which is not possible with a SISO radar system. For instance, if the scatterer moves in the direction of the boresight of ${\mathrm{Radar}}_{1}$, then ${\mathrm{Radar}}_{1}$ will detect the motion, while ${\mathrm{Radar}}_{2}$ may not. Conversely, ${\mathrm{Radar}}_{2}$ will obtain a relatively much stronger movement signature if the scatterer moves in the direction of the boresight of ${\mathrm{Radar}}_{2}$.

**Figure 2:**
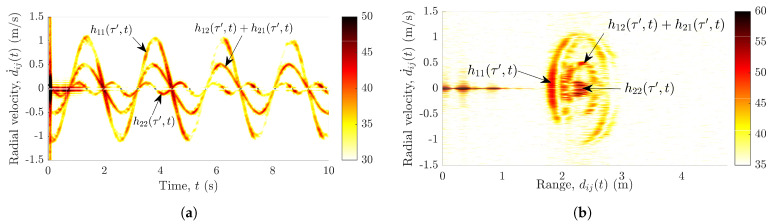
Radial velocity ${\dot{d}}_{ij}\left(t\right)$ of the pendulum vs. (**a**) time *t* and (**b**) range $d_{ij}\left(t\right)$ for the measured subchannel ${\tilde{h}}_{22}\left(\tau^{\prime },t\right)$.

The pendulum is set to swing in a direction parallel to the boresight of ${\mathrm{Radar}}_{1}$. The pendulum’s trajectories are recorded simultaneously by two subradars. Then, the recorded raw data are processed and the spectrogram is computed individually for each radar unit. Section 4 provides the details on the computation of the spectrogram from the radar’s raw data. Subsequently, the radial velocity profile is computed from the spectrogram (see Section 4). The radial velocity profile of the measured TV-CIR ${\tilde{h}}_{22}\left(\tau^{\prime },t\right)$ in the presence of the swinging pendulum is shown in Figure 2a. Figure 2b shows the motion of the pendulum in terms of the radial velocity ${\dot{d}}_{ij}\left(t\right)$ and range $d_{ij}\left(t\right)$. Although both subradars experience interferences, for brevity, only the measurements from ${\mathrm{Radar}}_{2}$ are shown here in Figure 2.

Evidently, the radial velocity profile in Figure 2a not only contains the pendulum’s trajectories from the desired wireless link $A_{2}^{T_{x}}$–$A_{2}^{R_{x}}$, but also the undesired trajectories from the interfering links $A_{1}^{T_{x}}$–$A_{1}^{R_{x}}$, $A_{1}^{T_{x}}$–$A_{2}^{R_{x}}$, and $A_{2}^{T_{x}}$–$A_{1}^{R_{x}}$. Similarly, Figure 2b also aids unmasking the problem of interference by depicting the three separate curves corresponding to the links $A_{i}^{T_{x}}$–$A_{j}^{R_{x}}$. As expected, the radial velocities of the pendulum in Figure 2a,b are identical for the links $A_{1}^{T_{x}}$–$A_{2}^{R_{x}}$ and $A_{2}^{T_{x}}$–$A_{1}^{R_{x}}$. In Figure 2a,b, the three different components of the swinging pendulum are labeled with the corresponding TV-CIRs $h_{ij}\left(\tau^{\prime },t\right)$. Furthermore, we have confirmed and validated this observed phenomenon of cross-channel interference by simulating the different wireless links $A_{i}^{T_{x}}$–$A_{j}^{R_{x}}$. The geometrical 3D indoor channel model and the extended pendulum model have been presented in Section 3 and Section 6, respectively, enabling the simulation of the wireless links $A_{i}^{T_{x}}$–$A_{j}^{R_{x}}$.

The aforementioned interferences encountered by the MIMO radar system hinder us to track the scatterer’s motion. To efficiently compute the radial range and radial velocity of the scatterer at each radar, we must first eradicate the interferences shown in Figure 2. This impels us to propound a solution to the problem of cross-channel interferences, which is presented in Section 5. For a better understanding of the proposed solution, we first describe the underlying geometrical 3D indoor model and the radar system model in Section 3 and Section 4, respectively.

## 3. Geometrical 3D Indoor Channel Model

**Figure 3:**
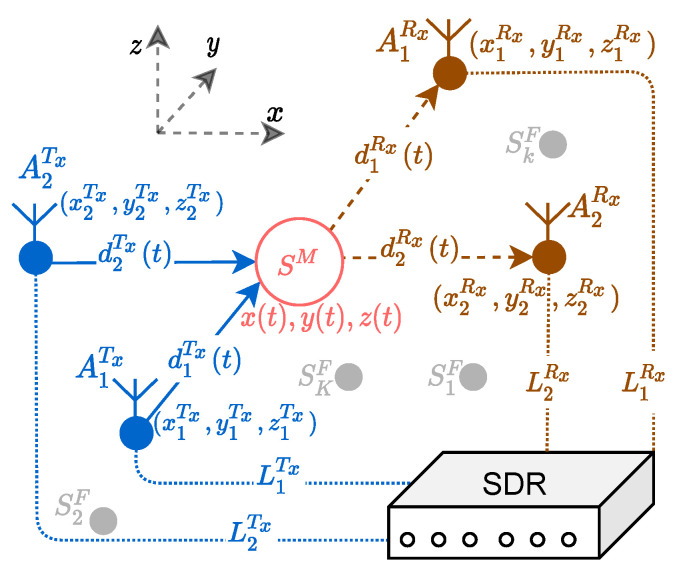
Geometrical 3D model for a 2×2 MIMO system with a single moving scatterer $S^{M}$ and *K* fixed scatterers $S_{k}^{F}$ (k=1,2,⋯,K).

In this section, we consider a 2×2 MIMO system deployed in an indoor 3D propagation scenario as depicted in Figure 3. The transmitter antenna $A_{i}^{T_{x}}$ is placed at a fixed position $\left(x_{i}^{T_{x}},y_{i}^{T_{x}},z_{i}^{T_{x}}\right)$ for i=1,2. Similarly, the receiver antenna $A_{j}^{R_{x}}$ is fixed at the position $\left(x_{j}^{R_{x}},y_{j}^{R_{x}},z_{j}^{R_{x}}\right)$ for j=1,2. The RF cable of length $L_{i}^{T_{x}}$ ($L_{j}^{R_{x}}$) connects the *i*th transmitter (*j*th receiver) antenna to the SDR as illustrated in Figure 3. The 3D propagation scenario consists of a single moving object, which is modeled as a scatterer $S^{M}$ with the TV coordinates (x(t),y(t),z(t)) as shown in Figure 3. In addition, the propagation environment consists of *K* fixed objects $S_{k}^{F}$ (k=1,2,⋯,K), such as walls, furniture, and decoration items. As the fixed scatterers $S_{k}^{F}$ are of no interest, they are eliminated from the spectrogram by radar signal preprocessing techniques.

The TV trajectory C(t) of the moving scatterer $S^{M}$, the position $C_{i}^{T_{x}}$ of the transmitter antenna $A_{i}^{T_{x}}$, and the position $C_{j}^{R_{x}}$ of the receiver antenna $A_{j}^{R_{x}}$ are defined as

(3) $$C\left(t\right)={\left[\begin{array}{ccc} x(t) & y(t) & z(t) \end{array}\right]}^{T}$$

(4) $$C_{i}^{T_{x}}={\left[\begin{array}{ccc} x_{i}^{T_{x}} & y_{i}^{T_{x}} & z_{i}^{T_{x}} \end{array}\right]}^{T}$$

and

(5) $$C_{j}^{R_{x}}={\left[\begin{array}{ccc} x_{j}^{R_{x}} & y_{j}^{R_{x}} & z_{j}^{R_{x}} \end{array}\right]}^{T}$$

respectively. The Euclidean distance between the *i*th transmitter (*j*th receiver) antenna and the non-stationary scatterer $S^{M}$ is denoted by $d_{i}^{T_{x}}\left(t\right)$ and $d_{j}^{R_{x}}\left(t\right)$, which can be expressed as

(6) $$d_{i}^{T_{x}}\left(t\right)=∥\begin{array}{c} C\left(t\right)-C_{i}^{T_{x}} \end{array}∥$$

and

(7) $$d_{j}^{R_{x}}\left(t\right)=∥\begin{array}{c} C\left(t\right)-C_{j}^{R_{x}} \end{array}∥$$

respectively, where $∥\begin{array}{c} x \end{array}∥$ denotes the Euclidean norm of *x*. The TV radial velocity components ${\dot{d}}_{i}^{T_{x}}\left(t\right)$ and ${\dot{d}}_{j}^{R_{x}}\left(t\right)$ can be represented as

(8) $${\dot{d}}_{i}^{T_{x}}\left(t\right)=\frac{1}{d_{i}^{T_{x}}\left(t\right)}{\left[\begin{array}{c} \dot{C}\left(t\right) \end{array}\right]}^{T}\left[\begin{array}{c} C\left(t\right)-C_{i}^{T_{x}} \end{array}\right]$$

and

(9) $${\dot{d}}_{j}^{R_{x}}\left(t\right)=\frac{1}{d_{j}^{R_{x}}\left(t\right)}{\left[\begin{array}{c} \dot{C}\left(t\right) \end{array}\right]}^{T}\left[\begin{array}{c} C\left(t\right)-C_{j}^{R_{x}} \end{array}\right]$$

respectively. The radar’s radial range $d_{ij}\left(t\right)$ of the moving scatterer $S^{M}$ is given by 1/2 of the total propagation distance, i.e.,

(10) $$d_{ij}\left(t\right)=\frac{1}{2}\left[d_{i}^{T_{x}}\left(t\right)+d_{j}^{R_{x}}\left(t\right)+L_{i}^{T_{x}}+L_{j}^{R_{x}}\right].$$

Finally, the composite radial velocity ${\dot{d}}_{ij}\left(t\right)$ can be expressed as

(11) $${\dot{d}}_{ij}\left(t\right)=\frac{1}{2}\left[{\dot{d}}_{i}^{T_{x}}\left(t\right)+{\dot{d}}_{j}^{R_{x}}\left(t\right)\right].$$

## 4. Radar System Model

For a 2×2 MIMO TDMA FMCW radar system, the transmitter signal $s_{i}\left(t^{\prime }\right)$ is defined as

(12) $$s_{i}\left(t^{\prime }\right)=\exp\left[j\phi_{i}+j2\pi \left(\frac{c_{r}}{2}t^{\prime 2}+f_{0}t^{\prime }\right)\right]$$

for i=1,2, where $\phi_{i}$ is the initial phase, $c_{r}$ is the chirp rate, and $f_{0}$ is the start frequency. The chirp rate $c_{r}$ is defined as $c_{r}=\left(f_{1}-f_{0}\right)/T_{sw}$, where $f_{1}$ is the stop frequency, and $T_{sw}$ is the sweep time of the periodic up-chirp signal being transmitted. In the TDMA mode, both transmitters operate in different time slots but use the same waveform as in (12). The time slots for the *i*th transmitter are defined as $\left(2n+i-1\right)T_{sw}\leq t^{\prime }<\left(2n+i\right)T_{sw}$ for n=0,1,⋯.

The transmitted signal $s_{i}\left(t^{\prime }\right)$ is reflected to the radar receiver antennas due to stationary and non-stationary scatterers present in the indoor environment. Therefore, each multipath component associated with the link $A_{i}^{T_{x}}$–$A_{j}^{R_{x}}$ experiences a propagation delay $\tau_{ij}^{\prime (l)}$ for l=1,2,⋯,L, where L denotes the total number of scatterers, which is given by L=K+1. The received signal, which is modeled as a weighted sum of L back-scattered multipath components, is then passed through the quadrature mixer stage of the radar. At the output of the mixer, we obtain the so-called beat (also known as deramped, dechirped or intermediate frequency) signal. The beat signal $s_{b,ij}^{\left(l\right)}\left(t^{\prime }\right)$ corresponding to the channel link $A_{i}^{T_{x}}$–$A_{j}^{R_{x}}$ in the presence of a particular scatterer $S^{\left(l\right)}$ is given as [38]

(13) $$s_{b,ij}^{\left(l\right)}\left(t^{\prime }\right)=a_{ij}^{\left(l\right)}\exp\left(j2\pi f_{b,ij}^{\left(l\right)}t^{\prime }+j\phi_{ij}^{\left(l\right)}\right)$$

where

(14) $$f_{b,ij}^{\left(l\right)}=\frac{2d_{ij}^{\left(l\right)}c_{r}}{c_{0}}$$

is the beat frequency, and

(15) $$\phi_{ij}^{\left(l\right)}=\frac{4\pi d_{ij}^{\left(l\right)}}{\lambda }$$

is the phase corresponding to the range $d_{ij}^{\left(l\right)}=c_{0}\tau_{ij}^{\prime (l)}/2$, where $c_{0}$ is the speed of light, and λ is the radar’s wavelength. The symbol $a_{ij}^{\left(l\right)}$ in (13) represents the net amplitude attenuation, which is related to the radar cross section of the *l*th scatterer, antenna gains, and transmission losses. In the presence of L scatterers in the radar’s field of view (FOV), the composite beat signal $s_{b,ij}\left(t^{\prime }\right)$ is simply the sum of all beat signals, i.e.,

(16) $$s_{b,ij}\left(t^{\prime }\right)=\sum_{l=1}^{L}s_{b,ij}^{\left(l\right)}\left(t^{\prime }\right).$$

Furthermore, note that according to the authors of [39], the complex conjugate of the composite beat signal $s_{b,ij}^{*}\left(t^{\prime }\right)$ is equal to the Fourier transform of the TV-CIR $h_{ij}\left(\tau^{\prime },t\right)$, i.e.,

(17) $$s_{b,ij}^{*}\left(t^{\prime }\right)=F\left\{h_{ij}\left(\tau^{\prime },t\right)\right\}$$

where F represents the Fourier transform. The time delay $\tau^{\prime }$ in (17) is related to the dual value of $t^{\prime }$ denoted by $f_{b}$ as $\tau^{\prime }=f_{b}/c_{r}$. Due to relation (17) and F{.} being a linear operator, the interference components in (1) and (2) also affect the measured composite beat signal $s_{b,ij}\left(t^{\prime }\right)$.

The composite beat signal $s_{b,ij}\left(t^{\prime }\right)$ is sampled by an analog-to-digital converter (ADC) module with sampling frequency $F_{s}\;=\;1/T_{s}$, where $T_{s}$ is the sampling interval. Let $N_{s}$ denote the number of samples taken from $s_{b,ij}\left(t^{\prime }\right)$ with the sampling interval $T_{sw}$, and let $N_{c}$ denote the number of chirps within a frame of the FMCW radar. Then, for a single frame duration of $T_{f}=N_{c}\times N_{s}\times T_{s}$, the sampled beat signal $s_{b,ij}\left(nT_{s}\right)$ can be arranged in a raw data matrix $D_{ij}$ as

(18) $$D_{ij}=\begin{array}{cc} \; & \left[\begin{array}{cccc} s_{b,ij}\left(0\right) & s_{b,ij}\left(T_{s}\right) & \cdots & s_{b,ij}\left(T_{sw}-T_{s}\right)\\ s_{b,ij}\left(T_{sw}\right) & s_{b,ij}\left(T_{sw}+T_{s}\right) & \cdots & s_{b,ij}\left(2T_{sw}-T_{s}\right)\\ \vdots & \vdots & \vdots & \vdots \\ s_{b,ij}\left(\left(N_{c}-1\right)T_{sw}\right) & s_{b,ij}\left(\left(N_{c}-1\right)T_{sw}+T_{s}\right) & \cdots & s_{b,ij}\left(N_{c}T_{sw}-T_{s}\right) \end{array}\right. \end{array}$$

where $T_{sw}=N_{s}T_{s}$. Note that the dimension of the raw data matrix is $N_{c}\times N_{s}$. Each row of $D_{ij}$ contains the fast-time data that has been sampled with the sampling interval $T_{s}$, and each column of $D_{ij}$ contains the slow-time data sampled with the sampling interval $T_{sw}$.

The fast Fourier transform (FFT) of the fast-time data is known as the range FFT. The range FFT is applied to the rows of the raw data matrix $D_{ij}$ to acquire the beat frequencies $f_{b,ij}^{\left(l\right)}$ of the composite beat signal $s_{b,ij}\left(t^{\prime }\right)$ (see (13)). Subsequently, the range maps or the range $d_{ij}^{\left(l\right)}$ for each scatterer can be computed using the relation in (14). As the observation interval of the range FFT is $T_{sw}$, the frequency resolution $f_{\mathrm{res}}$ of the range FFT is limited to $f_{\mathrm{res}}=1/T_{sw}$. Therefore, it can be shown [40] that the spectral components caused by two different moving scatterers at different ranges can be resolved in the spectrum of (16) provided that the scatterers are at least

(19) $$d_{\mathrm{res}}=\frac{c_{0}}{2B}$$

apart in range, where $d_{\mathrm{res}}$ is the range resolution, and *B* is the bandwidth of the radar. Furthermore, from the Nyquist criterion, it can be shown [41] that the radar’s maximum unambiguous range is $d_{\max}=F_{s}c_{0}/2c_{r}$.

Let us define $\Delta d_{ij}^{\left(l\right)}$, $\Delta \tau_{ij}^{\prime (l)}$, $\Delta \phi_{ij}^{\left(l\right)}$, and $\Delta f_{b,ij}^{\left(l\right)}$ as the net change in $d_{ij}^{\left(l\right)}$, $\tau_{ij}^{\prime (l)}$, $\phi_{ij}^{\left(l\right)}$, and $f_{b,ij}^{\left(l\right)}$, respectively, over the period of one sweep interval $T_{sw}$. Note that a moving scatterer is fixed over an observation window $T_{sw}$, because $\Delta d_{ij}^{\left(l\right)}\ll d_{\mathrm{res}}$. Therefore, a small change in the displacement $\Delta d_{ij}^{\left(l\right)}$ results in a small change in the frequency of the beat signal, denoted by $\Delta f_{b,ij}^{\left(l\right)}$. This frequency change $\Delta f_{b,ij}^{\left(l\right)}$ is not discernible in the spectrum of (16) because $\Delta f_{b,ij}^{\left(l\right)}<f_{\mathrm{res}}$. In order to capture $\Delta d_{ij}^{\left(l\right)}$, we need to observe the phase of the beat signal $\phi_{ij}^{\left(l\right)}$ over multiple sweep intervals $T_{sw}$. The phase of the beat signal is very sensitive and changes significantly from sweep to sweep even for slight displacements of the scatterer. In analogy to (15), the relation of the phase change $\Delta \phi_{ij}^{\left(l\right)}$ and the displacement $\Delta d_{ij}$ is given as

(20) $$\Delta \phi_{ij}^{\left(l\right)}=\frac{4\pi \Delta d_{ij}^{\left(l\right)}}{\lambda }.$$

Therefore, the phase change $\Delta \phi_{ij}^{\left(l\right)}$ of the beat signal can be observed over two sweeps to determine the radial velocity by means of

(21) $$v_{ij}^{\left(l\right)}=\frac{\lambda \Delta \phi_{ij}^{\left(l\right)}}{4\pi T_{sw}}.$$

However, two or more equidistant scatterers with different radial velocities cannot be resolved using the phase difference observed only over two chirps. To capture all the different phase changes $\Delta \phi_{ij}^{\left(l\right)}$ corresponding to the equidistant non-stationary scatterers, the Doppler FFT is applied to the columns of the radar range maps to obtain the micro-Doppler frequencies $f_{d,ij}^{\left(l\right)}\left(t\right)$. From the micro-Doppler frequencies $f_{d,ij}^{\left(l\right)}\left(t\right)$, the radial velocities $v_{ij}^{\left(l\right)}\left(t\right)$ can be computed as

(22) $$v_{ij}^{\left(l\right)}\left(t\right)=\frac{f_{d,ij}^{\left(l\right)}\left(t\right)c_{0}}{2f_{0}}.$$

Furthermore, the radar velocity resolution is given as $v_{\mathrm{res}}=\lambda /2T_{f}$. The maximum unambiguous radial velocity can be derived as $v_{\max}=\lambda /4T_{sw}$.

The components of the radar signal processing of the raw data matrix $D_{ij}$ are delineated here. First, the Hanning window function

(23) $$w_{H}\left(t^{\prime }\right)=\left\{\begin{array}{cc} \frac{1}{2}\left[1-\cos\left(\frac{2\pi t^{\prime }}{T_{sw}}\right)\right], & 0\leq t^{\prime }\leq T_{sw}\\ 0, & \mathrm{otherwise} \end{array}\right.$$

is applied to the fast-time data of the frame, where the window length is equal to the chirp duration $T_{sw}$. Then, the range maps are computed by applying the range FFT to the windowed data. To acquire the range evolution of the scatterers over time, the slow-time data can be agglomerated to obtain the processing gain.

After the application of the range FFT, the slow-time data are split into many overlapping or consecutive disjoint segments. Then, for each segment and each range-bin, the short-time Doppler FFT is computed to obtain the local micro-Doppler information of the scatterers. A further processing gain can be achieved by agglomerating the range maps. In other words, for a particular range, the slow-time non-stationary data are composed of the TV micro-Doppler frequencies of the scatterers, which can be obtained by the spectrogram defined as [42]

(24) $$S_{ij}\left(f,t\right)={\left|\int_{-\infty }^{\infty }x_{ij}\left(t^{\prime \prime },t\right)e^{-j2\pi ft^{\prime \prime }}dt^{\prime \prime }\right|}^{2}$$

where

(25) $$x_{ij}\left(t^{\prime \prime },t\right)=s_{b,ij}\left(t^{\prime \prime }\right)w_{R}\left(t^{\prime \prime }-t\right)$$

in which *t* is the local time, and $t^{\prime \prime }$ represents the running time. In (25), $w_{R}\left(t^{\prime \prime }\right)$ denotes a window function, which is in our case a rectangular function defined as

(26) $$w_{R}\left(t^{\prime \prime }\right)=\left\{\begin{array}{cc} 1, & 0\leq t^{\prime \prime }<N_{c}T_{sw}\\ 0, & \mathrm{otherwise}. \end{array}\right.$$

Finally, from the spectrogram $S_{ij}\left(f,t\right)$, we can compute the TV mean Doppler shift as

(27) $$B_{ij}^{\left(1\right)}\left(t\right)=\frac{\int_{-\infty }^{\infty }fS_{ij}\left(f,t\right)df}{\int_{-\infty }^{\infty }S_{ij}\left(f,t\right)df}.$$

The measured mean Doppler shift $B_{ij}^{\left(1\right)}\left(t\right)$ will be compared with the mean Doppler shift of the analytical model in Section 6 for the cross-validation of the experimental results and the analytical results.

## 5. Proposed Solution

**Figure 4:**
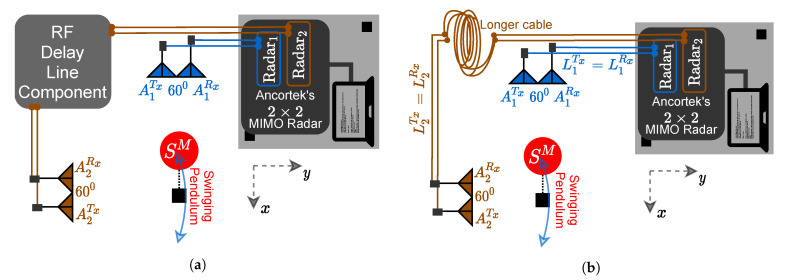
Setup to induce a fixed propagation delay by either using (**a**) an RF delay line component or (**b**) different cable lengths, i.e., $\left(L_{1}^{T_{x}},L_{1}^{R_{x}}\right)\neq \left(L_{2}^{T_{x}},L_{2}^{R_{x}}\right)$.

In this section, we propose a solution to mitigate the problem of the cross-channel interferences described in Section 2. The proposed approach is to induce a controlled propagation delay in one of the subchannels, so that the desired channel links $A_{1}^{T_{x}}$–$A_{1}^{R_{x}}$ and $A_{2}^{T_{x}}$–$A_{2}^{R_{x}}$ can be separated in the range domain of the MIMO radar. To this end, we can use an RF delay line component as a tool for increasing the propagation delay in one of the subradars of the 2×2 MIMO radar system shown in Figure 4a. More conveniently, a pair of RF cables with different lengths can be used instead of the RF delay line component to induce a fixed propagation delay in the channel of interest as shown in Figure 4b. As illustrated in Figure 3, a cable of length $L_{i}^{T_{x}}$ connects the SDR to the *i*th transmitter antenna $A_{i}^{T_{x}}$, and a cable of length $L_{j}^{R_{x}}$ connects the SDR to the *j*th receiver antenna $A_{j}^{R_{x}}$.

For each subradar, the cables of the same length are used for the transmitter and the receiver antennas, i.e., $L_{i}^{T_{x}}=L_{j}^{R_{x}}$ for i=j. To obtain a virtual propagation delay in the link $A_{2}^{T_{x}}$–$A_{2}^{R_{x}}$, we choose the cable lengths $L_{2}^{T_{x}}$ and $L_{2}^{R_{x}}$ depending on the dimensions of the indoor environment or the desired coverage area of the MIMO radar system. We deploy connector cables with lengths $L_{2}^{T_{x}}$ and $L_{2}^{R_{x}}$ according to the relations

(28) $$L_{2}^{T_{x}}\geq 2L_{a}+L_{1}^{T_{x}}$$

and

(29) $$L_{2}^{R_{x}}\geq 2L_{a}+L_{1}^{R_{x}}$$

respectively, where $L_{a}$ represents the length of the area of interest, which is essentially the square area covered by the MIMO radar system. Using (28) and (29), the channel links $A_{i}^{T_{x}}$–$A_{j}^{R_{x}}$ are guaranteed to be separable for the scatterers in the square area $A_{sq}=L_{a}\cdot L_{a}$. Therefore, the radar range $d_{ij}\left(t\right)$ in (10) is controlled using a longer pair of cables for the link $A_{2}^{T_{x}}$–$A_{2}^{R_{x}}$. Then, the radial ranges of the channel links $A_{i}^{T_{x}}$–$A_{j}^{R_{x}}$ follow the inequality $d_{11}\left(t\right)\;<\;d_{12}\left(t\right)\;<\;d_{22}\left(t\right)$. Furthermore, the links $A_{1}^{T_{x}}$–$A_{2}^{R_{x}}$ and $A_{2}^{T_{x}}$–$A_{1}^{R_{x}}$ have identical radial distances, i.e., $d_{12}\left(t\right)\;=\;d_{21}\left(t\right)$.

Finally, an additional range gating module is implemented after the range FFT module in the radar signal processing chain described in Section 4. The range profile of the MIMO radar system (obtained by the range FFT module) is partitioned by the range gating module to acquire $d_{11}\left(t\right)$, $d_{22}\left(t\right)$, and $d_{12}\left(t\right)$. In other words, the range gating module segregates the independent trajectories of the scatterers for each channel link $A_{i}^{T_{x}}$–$A_{j}^{R_{x}}$. Subsequently, each channel link can now be further processed without the problem of cross-channel interferences. The results of the proposed approach are presented in the subsequent section.

Note that the proposed approach can also be adopted to completely avoid the use of the TDMA scheme. The TDMA scheme limits the PRF of the MIMO radar system, which in turn limits the system’s maximum measurable unambiguous radial velocity $v_{\max}$. The PRF and the maximum radial velocity $v_{\max}$ decrease by the same factor as the number of subradars of the MIMO system increases. On the other hand, the proposed approach allows multiple RF delay lines to be used for different channel links $A_{i}^{T_{x}}$–$A_{j}^{R_{x}}$ so that all the subradars can operate simultaneously without effecting the PRF and $v_{\max}$ of the MIMO radar system. For instance, for an N×N MIMO radar system, the cable difference for different channel links $A_{i}^{T_{x}}$–$A_{j}^{R_{x}}$ must follow the inequality $\min\left[L_{i}^{T_{x}/R_{x}}-L_{j}^{T_{x}/R_{x}}\right]\;\geq 2L_{a}$ for i≠j, where i,j∈{1,2,⋯,N}.

## 6. Experimental Results

In this section, we elaborate our measurement campaign carried out using an FMCW-based MIMO radar system (Ancortek SDR-KIT 2400T2R4) operating in the K-band. The detailed analytical model for a swinging pendulum is laid out in this section for the validation of the experimental results. The efficacy of the proposed solution against the interferences of Ancortek’s MIMO radar system is also highlighted by the measurement results.

The measurements were carried out in a semi-controlled environment, a laboratory with the dimensions of 11.5m×6m. The laboratory was equipped with many stationary objects such as chairs, tables, boards, and computers. The pendulum bob weighing 3 kg was suspended from the ceiling of the laboratory by means of a rope of length *L*. The pendulum bob acted as a single non-stationary scatterer (L=K+1) initially resting at the coordinates (0,0,1.07)m. The Ancortek radar was placed inside the laboratory and configured as a 2×2 MIMO radar system in FMCW mode. The transmitter antennas $A_{1}^{T_{x}}$ and $A_{2}^{T_{x}}$, and the receiver antennas $A_{1}^{R_{x}}$ and $A_{2}^{R_{x}}$ were positioned in a monostatic configuration according to Table 1. The length of the RF cables, $L_{i}^{T_{x}}$ and $L_{j}^{R_{x}}$, the maximum displacement $x_{\max}$ and the length *L* of the pendulum, and the MIMO radar operating parameters $f_{c}$, BW, $T_{sw}$, and PRF were fixed according to the values listed in Table 1. The two subradars of the MIMO system were configured to share the time according to the TDMA scheme, but even so, the Ancortek system experienced cross-channel interference as stated in Section 2. Needless to say, due to the TDMA mode of operation, the PRF of the subradars was reduced to half, i.e., $\mathrm{PRF}=1/2T_{sw}$, as listed in Table 1.

We now present the analytical model for the pendulum swinging in *xz*-plane, so that we are able to cross-validate the experimental results with the analytical results. The pendulum is displaced by $x_{\max}$ to set it in a swinging motion. The TV nonlinear trajectories of the pendulum can be obtained as [43]

(30) $$x\left(t\right)=L\sin\left\{\arcsin\left(\frac{x_{\max}}{L}\right)\cos\left(\sqrt{\frac{g}{L}}t\right)\right\}$$

(31) y(t)=0

(32) $$z\left(t\right)=L\left[1-\cos\left\{\arcsin\left(\frac{x(t)}{L}\right)\right\}\right]$$

where *g* represents the gravitational field strength. The above model for the pendulum’s trajectories is valid for an ideal pendulum, which swings only in the *xz*-plane. The model can readily be used for a pendulum swinging in the *yz*-plane by interchanging the right-hand side of the expressions in (30) and (31). To analytically determine the radial range of the scatterer, the pendulum model expressed by (30)–(32) can be used with (10) of the geometrical 3D indoor channel model introduced in Section 3. On the other hand, to obtain the radial velocity using (11), we must first derive the expressions for $\dot{x}\left(t\right)$, $\dot{y}\left(t\right)$, and $\dot{z}\left(t\right)$, which results in

(33) $$\dot{x}\left(t\right)=-\sqrt{Lg}\cos\left(\phi^{\prime }\right)\arcsin\left(\frac{x_{\max}}{L}\right)\sin\left(\sqrt{\frac{g}{L}}t\right)$$

(34) $$\dot{y}\left(t\right)=0$$

and

(35) $$\dot{z}\left(t\right)=\frac{x\left(t\right)\dot{x}\left(t\right)}{\sqrt{L^{2}-x^{2}\left(t\right)}}\;,\;\left|x(t)\right|\leq L$$

respectively, where $\phi^{\prime }=\arcsin\left(x_{\max}/L\right)\cos\left(\sqrt{g/L}\cdot t\right)$. By making use of the extended pendulum model (30)–(35) combined with the geometrical 3D indoor channel model, we can compute analytically the TV radial range components $d_{ij}\left(t\right)$ and the radial velocity components ${\dot{d}}_{ij}\left(t\right)$ for all wireless channel links $A_{i}^{T_{x}}$–$A_{j}^{R_{x}}$ shown in Figure 1.

**Figure 5:**
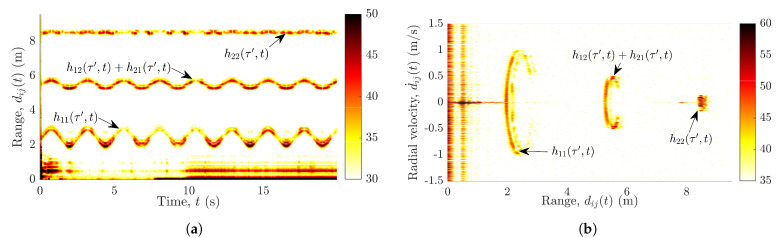
Different cable lengths, i.e., $\left(L_{1}^{T_{x}},L_{1}^{R_{x}}\right)\neq \left(L_{2}^{T_{x}},L_{2}^{R_{x}}\right)$, result in the segregation of (**a**) measured range profiles and (**b**) measured range–velocity profiles.

For the experimental setup from Table 1, the measured radial range profile is shown in Figure 5a and the measured radial velocity profile is plotted against the measured range in Figure 5b. The two subradars capture and process the nonlinear trajectories of the pendulum by means of the radar signal preprocessing described in Section 4. We obtain the processing gain in the radial range profile by agglomerating the slow-time data, whereas the radial velocity profile is acquired by integrating over the range maps. The radial range profile is obtained from the measured beat frequency profile by using (14). On the other hand, the radial velocity profile is mapped from the measured micro-Doppler frequency profile by utilizing the relation in (22). The two subradars adopt the proposed solution (see Section 5) for the mitigation of the cross-channel interferences encountered by the Ancortek MIMO radar system. Figure 5a,b illustrates the effect of different cable lengths on the measured range profile for a pendulum swinging in the *xz*-plane. Due to the deployment of different cables, three distinct curves can be observed in Figure 5a,b that can be segregated by means of the range gating module (see Section 5). While the pendulum swings in the *xz*-plane, the radial range $d_{11}\left(t\right)$ in Figure 5a changes to a much greater extent than the radial ranges $d_{22}\left(t\right)$ and $d_{12}\left(t\right)$ or $d_{21}\left(t\right)$. A similar inference can be drawn regarding the radial velocities ${\dot{d}}_{ij}\left(t\right)$ in Figure 5b.

**Figure 6:**
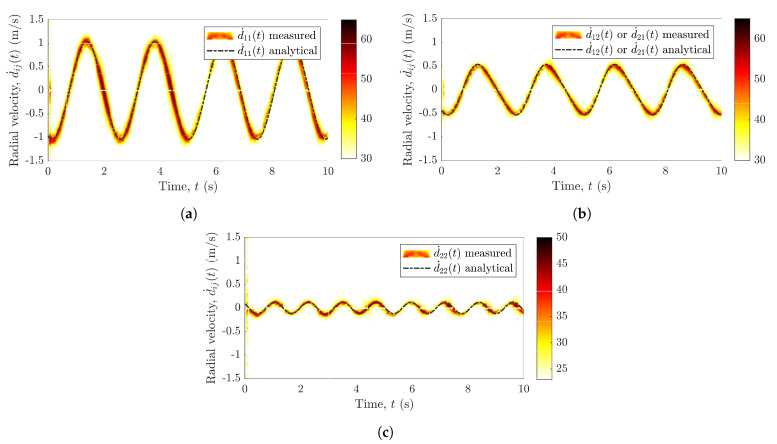
Application of the proposed interference mitigation scheme results in segregated measured radial velocity components ${\dot{d}}_{ij}\left(t\right)$ for the channel links: (**a**) $A_{1}^{T_{x}}$–$A_{1}^{R_{x}}$ (${\mathrm{Radar}}_{1}$), (**b**) $A_{1}^{T_{x}}$–$A_{2}^{R_{x}}$ (or $A_{2}^{T_{x}}$–$A_{1}^{R_{x}}$), and (**c**) $A_{2}^{T_{x}}$–$A_{2}^{R_{x}}$ (${\mathrm{Radar}}_{2}$).

After the application of the proposed interference mitigation approach, we obtain the distinct radial velocity components ${\dot{d}}_{11}\left(t\right)$, ${\dot{d}}_{12}\left(t\right)$ or ${\dot{d}}_{21}\left(t\right)$, and ${\dot{d}}_{22}\left(t\right)$ as illustrated in Figure 6a–c, respectively, where ${\dot{d}}_{12}\left(t\right)={\dot{d}}_{21}\left(t\right)$. The MIMO radar system captures the pendulum trajectories in the *x*-axis and *y*-axis, which signifies the importance of the deployment of multiple RF sensors in an indoor environment. Figure 6a,c depicts the radial velocities corresponding to ${\mathrm{Radar}}_{1}$ and ${\mathrm{Radar}}_{2}$, respectively, whereas Figure 6b shows the radial velocities corresponding to the channel link $A_{1}^{T_{x}}$–$A_{2}^{R_{x}}$ or $A_{2}^{T_{x}}$–$A_{1}^{R_{x}}$. The pendulum is swinging in the *xz*-plane (parallel to the boresight of ${\mathrm{Radar}}_{1}$), consequently, one can observe that the radial velocity is much higher in Figure 6a compared to Figure 6c. Furthermore, as anticipated, the number of crests and troughs in the radial velocity profile of ${\mathrm{Radar}}_{2}$ is twice as high. Note that the radial velocities ${\dot{d}}_{11}\left(t\right)$ and ${\dot{d}}_{22}\left(t\right)$ captured by ${\mathrm{Radar}}_{1}$ and ${\mathrm{Radar}}_{2}$, respectively, are independent and unique, which cannot be achieved with a SISO system. Moreover, the measured radial velocities are validated by the analytical model that comprises the geometrical 3D indoor model for the distributed MIMO system (see Section 3) and the extended pendulum model described by (30)–(35). A good match between the measurements and the analytical model is shown in Figure 6, which confirms the validity of the geometrical 3D indoor model and the extended pendulum model. The efficacy of the proposed approach against the interferences can be apprehended by comparing Figure 6 with Figure 2a. Evidently, the proposed approach eliminates the cross-channel interferences altogether by separating the measured trajectories for each radar of the MIMO system. Therefore, although the radial velocity components in Figure 6 are identical to the radial velocity components of Figure 2a, they are without any interferences.

**Figure 7:**
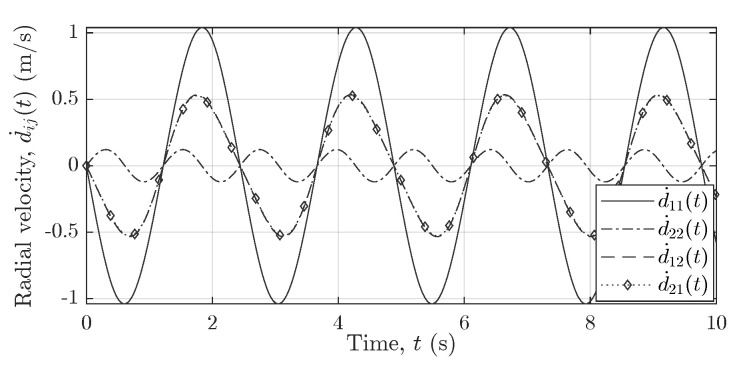
Analytical radial velocity components ${\dot{d}}_{ij}\left(t\right)$ for the channel links $A_{1}^{T_{x}}$–$A_{1}^{R_{x}}$, $A_{1}^{T_{x}}$–$A_{2}^{R_{x}}$, $A_{2}^{T_{x}}$–$A_{1}^{R_{x}}$, and $A_{2}^{T_{x}}$–$A_{2}^{R_{x}}$.

**Figure 8:**
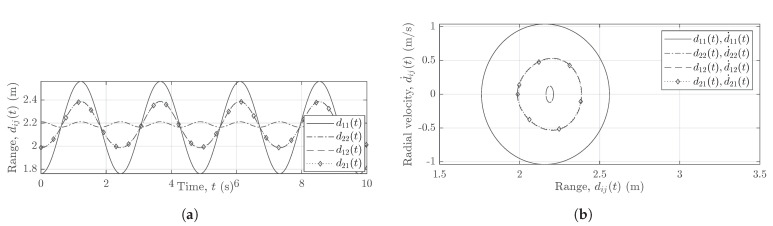
(**a**) The analytical radial range components $d_{ij}\left(t\right)$ and (**b**) the analytical radial velocity components ${\dot{d}}_{ij}\left(t\right)$ for $\left(L_{1}^{T_{x}},L_{1}^{R_{x}}\right)=\left(L_{2}^{T_{x}},L_{2}^{R_{x}}\right)$.

**Figure 9:**
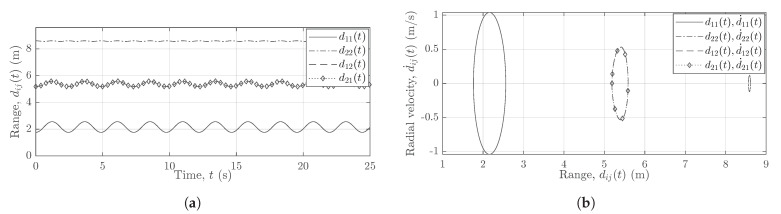
(**a**) The analytical radial range components $d_{ij}\left(t\right)$ and (**b**) the analytical radial velocity components ${\dot{d}}_{ij}\left(t\right)$ for $\left(L_{1}^{T_{x}},L_{1}^{R_{x}}\right)\neq \left(L_{2}^{T_{x}},L_{2}^{R_{x}}\right)$.

Figure 7, Figure 8 and Figure 9 show the reference curves for the nonlinear trajectories of the pendulum, which are used to cross-validate the measurement results obtained for all subchannel links $A_{i}^{T_{x}}$–$A_{j}^{R_{x}}$ of the 2×2 MIMO system. Figure 7, Figure 8 and Figure 9 illustrate the trajectories of the pendulum swinging in the *xz*-plane (parallel to the boresight of ${\mathrm{Radar}}_{1}$) within the FOV of the two subradars. Figure 7 illustrates the analytical radial velocity components ${\dot{d}}_{ij}\left(t\right)$ that do not depend on the deployment of longer cables. Figure 8a,b shows the scenario when the two subradars of the MIMO system use the same cable lengths, i.e., $\left(L_{1}^{T_{x}},L_{1}^{R_{x}}\right)=\left(L_{2}^{T_{x}},L_{2}^{R_{x}}\right)$, whereas Figure 9a,b shows the case when the two subradars use different cable lengths, i.e., $\left(L_{1}^{T_{x}},L_{1}^{R_{x}}\right)\neq \left(L_{2}^{T_{x}},L_{2}^{R_{x}}\right)$. Figure 9, analogous to Figure 5, shows the effect of longer cable lengths $L_{2}^{T_{x}}$ and $L_{2}^{R_{x}}$ on the radial ranges $d_{ij}\left(t\right)$.

**Figure 10:**
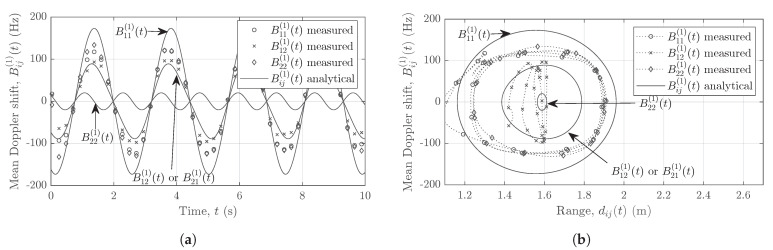
(**a**) The measured mean Doppler shift $B_{ij}^{\left(1\right)}\left(t\right)$ vs. time and (**b**) the measured mean Doppler shift $B_{ij}^{\left(1\right)}\left(t\right)$ vs. range $d_{ij}\left(t\right)$, where the MIMO radar undergoes cross-channel interferences.

The relation in (27) is utilized to obtain the measured mean Doppler shift $B_{ij}^{\left(1\right)}\left(t\right)$ for all channel links $A_{i}^{T_{x}}$–$A_{j}^{R_{x}}$ in a 2×2 MIMO system. Analogous to the computation of the mean Doppler shift, the mean radial range is obtained from the range profile. The analytical and measured mean Doppler shifts $B_{ij}^{\left(1\right)}\left(t\right)$ are illustrated in Figure 10a. Figure 10b shows the analytical and measured mean Doppler shifts plotted against the range of the moving scatterer $S^{M}$. Clearly, a considerable mismatch exists between the analytical and measured mean Doppler shifts due to the interferences.

**Figure 11:**
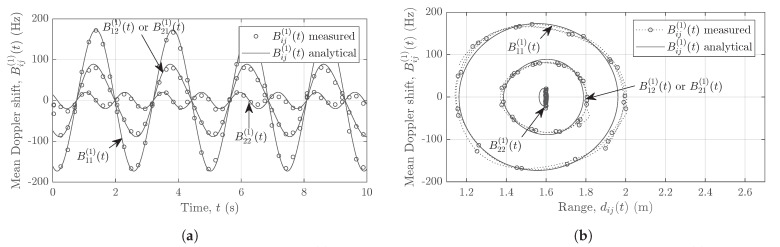
(**a**) The measured mean Doppler shift $B_{ij}^{\left(1\right)}\left(t\right)$ vs. time and (**b**) the measured mean Doppler shift $B_{ij}^{\left(1\right)}\left(t\right)$ vs. range $d_{ij}\left(t\right)$, where the MIMO radar adopts the proposed interference mitigation scheme.

On the other hand, using the proposed approach, we obtain the segregated nonlinear trajectories of the pendulum as shown in Figure 11. Figure 11a illustrates the mean Doppler shift of the pendulum swinging in the *xz*-plane over a period of 10 seconds. A good match between the measured and the analytical mean Doppler shifts is observed for all channel links $A_{i}^{T_{x}}$–$A_{j}^{R_{x}}$. Figure 11b shows the mean Doppler shift plotted against the mean radial range. Due to the fine Doppler resolution of the FMCW radar, the measured Doppler information matches very well with the analytical results in Figure 11, whereas an adequate match exists between the analytical and measured range due to an adequate range resolution of the system.

## 7. Conclusions

In this paper, we proposed a unique approach to the problem of cross-channel interferences encountered by the Ancortek SDR-KIT 2400T2R4 MIMO radar system due to its poor interchannel RF isolation. For all subchannels of the MIMO radar system, we observed a significant mismatch between the measured and analytical TV mean Doppler shift due to the problem of cross-channel interference. However, after the application of the proposed interference mitigation method, we found an excellent fit between the measured and analytical TV mean Doppler shift. The proposed approach is optimal and robust in a way that it completely eliminates the cross-channel interferences. The proposed solution works for the Ancortek MIMO radar system without the need to alter its firmware or hardware. We also presented a channel model to investigate the target’s motion in a MIMO system under different target–antenna configurations. A good agreement was found between the geometrical 3D indoor channel model and the measured data. In the proposed solution, the segregation and utilization of the cross-channel component generally lead to an added diversity and improved system capability. Although the proposed approach may find its utility in numerous application areas, we plan to extend this work to orientation-independent human activity recognition. For human activity recognition, we plan to fuse the data from different subchannels of the MIMO radar system to increase the overall classification performance of the system.

## Figures and Tables

**Table 1:** MIMO experimental setup.

Description	Parameters	Values
$A_{1}^{T_{x}}$ position	$\left(x_{1}^{T_{x}},y_{1}^{T_{x}},z_{1}^{T_{x}}\right)$	(1.56,0.01,1.195) m
$A_{1}^{R_{x}}$ position	$\left(x_{1}^{R_{x}},y_{1}^{R_{x}},z_{1}^{R_{x}}\right)$	(1.56,−0.01,1.185) m
$A_{2}^{T_{x}}$ position	$\left(x_{2}^{T_{x}},y_{2}^{T_{x}},z_{2}^{T_{x}}\right)$	(−0.01,1.56,1.195) m
$A_{2}^{R_{x}}$ position	$\left(x_{2}^{R_{x}},y_{2}^{R_{x}},z_{2}^{R_{x}}\right)$	(1.0,1.56,1.185) m
RF cable lengths	$\left(L_{1}^{T_{x}},L_{1}^{R_{x}},L_{2}^{T_{x}},L_{2}^{R_{x}}\right)$	(0.3,0.3,3.5,3.5) m
Length of pendulum	*L*	1.48 m
Max. displacement	$x_{\max}$	0.4 m
Carrier frequency	$f_{c}$	25 GHz
Radar’s bandwidth	BW	2 GHz
Sweep time	$T_{sw}$	1 ms
Pulse repetition freq.	PRF	500 Hz

## Data Availability

The authors may provide the presented measurement data upon request.

## References

[1] Lu X., Koga T. DAPS based adaptive tracking system for high-assurance air traffic surveillance. Proceedings of the 2014 Integrated Communications, Navigation and Surveillance Conference (ICNS) Conference Proceedings.

[2] Gómez-del Hoyo P.J., Del-Rey-Maestre N., Mata-Moya D., Jarabo-Amores M.P., Benito-Ortiz M.C. Coherent detection and 3D tracking stages of a DVB-T based passive radar for terrestrial traffic monitoring. Proceedings of the IOP Conference Series: Materials Science and Engineering.

[3] Nguyen D.A., Cho B., Seo C., Park J., Lee D.H. (2018). Analysis of the optimal frequency band for a ballistic missile defense radar system. J. Electromagn. Eng. Sci..

[4] Bandini F., Sunding T.P., Linde J., Smith O., Jensen I.K., Köppl C.J., Butts M., Bauer-Gottwein P. (2020). Unmanned Aerial System (UAS) observations of water surface elevation in a small stream: Comparison of radar altimetry, LIDAR and photogrammetry techniques. Remote Sens. Environ..

[5] Watts S. The ASV 21 maritime surveillance radar. Proceedings of the 2017 IEEE Radar Conference (RadarConf).

[6] Louf V., Protat A., Warren R.A., Collis S.M., Wolff D.B., Raunyiar S., Jakob C., Petersen W.A. (2019). An integrated approach to weather radar calibration and monitoring using ground clutter and satellite comparisons. J. Atmos. Ocean. Technol..

[7] Margot J.L. (2021). A Data-Taking System for Planetary Radar Applications. J. Astron. Instrum..

[8] Kranold L., Taherzadeh M., Nabki F., Coates M., Popovic M. (2021). Microwave breast screening prototype: System miniaturization with IC pulse radio. IEEE J. Electromagn. RF Microw. Med. Biol..

[9] Liu Z., Cai Y., Wang H., Chen L., Gao H., Jia Y., Li Y. (2021). Robust target recognition and tracking of self-driving cars with radar and camera information fusion under severe weather conditions. IEEE Trans. Intell. Transp. Syst..

[10] Waldschmidt C., Hasch J., Menzel W. (2021). Automotive Radar—From First Efforts to Future Systems. IEEE J. Microw..

[11] Uysal F. (2020). Phase-coded FMCW automotive radar: System design and interference mitigation. IEEE Trans. Veh. Technol..

[12] Frigeri A., Ercoli M. (2020). The ScanMars subsurface radar sounding experiment on AMADEE-18. Astrobiology.

[13] Du H., Jin T., He Y., Song Y., Dai Y. (2020). Segmented convolutional gated recurrent neural networks for human activity recognition in ultra-wideband radar. Neurocomputing.

[14] Li X., He Y., Jing X. (2019). A survey of deep learning-based human activity recognition in radar. Remote Sens..

[15] Luo F., Poslad S., Bodanese E. (2019). Human activity detection and coarse localization outdoors using micro-Doppler signatures. IEEE Sens. J..

[16] Jokanovic B., Amin M. (2018). Fall detection using deep learning in range-Doppler radars. IEEE Trans. Aerosp. Electron. Syst..

[17] Lien J., Gillian N., Karagozler M.E., Amihood P., Schwesig C., Olson E., Raja H., Poupyrev I. (2016). Soli: Ubiquitous gesture sensing with millimeter wave radar. ACM Trans. Graph..

[18] Yeong D.J., Velasco-Hernandez G., Barry J., Walsh J. (2021). Sensor and sensor fusion technology in autonomous vehicles: A Review. Sensors.

[19] Van N.T.P., Tang L., Singh A., Minh N.D., Mukhopadhyay S.C., Hasan S.F. (2019). Self-identification respiratory disorder based on continuous wave radar sensor system. IEEE Access.

[20] Piotrowsky L., Jaeschke T., Kueppers S., Siska J., Pohl N. (2019). Enabling high accuracy distance measurements with FMCW radar sensors. IEEE Trans. Microw. Theory Tech..

[21] Lee D., Shaker G., Melek W. (2020). Imaging of human walking behind the obstacle utilizing pulsed radar technique in the C-band for military surveillance applications. J. Electr. Eng. Technol..

[22] Abuduaini A., Shiraki N., Honma N., Nakayama T., Iizuka S. Performance evaluation of multiple human-body localization using bistatic MIMO radar. Proceedings of the 2019 IEEE Asia-Pacific Microwave Conference (APMC).

[23] Waqar S., Yusaf H., Sana S., Waqas M., Siddiqui F.A. Reconfigurable monopulse radar tracking processor. Proceedings of the 15th International Bhurban Conference on Applied Sciences and Technology, IBCAST 2018.

[24] Bovenga F. (2020). Special issue “synthetic aperture radar (SAR) techniques and applications”. Sensors.

[25] Feng C., Jiang X., Jeong M.G., Hong H., Fu C.H., Yang X., Wang E., Zhu X., Liu X. (2021). Multitarget vital signs measurement with chest motion imaging based on MIMO radar. IEEE Trans. Microw. Theory Tech..

[26] Sana S., Waqar S., Yusaf H., Waqas M., Siddiqui F.A. Software defined digital beam forming processor. Proceedings of the 13th International Bhurban Conference on Applied Sciences and Technology, IBCAST 2016.

[27] Sadeghi M., Behnia F., Amiri R., Farina A. (2021). Target localization geometry gain in distributed MIMO radar. IEEE Trans. Signal Process..

[28] Zhang H., Liu W., Zhang Z., Lu W., Xie J. (2021). Joint target assignment and power allocation in multiple distributed MIMO Radar networks. IEEE Syst. J..

[29] Wang P., Li H. (2020). Target detection with imperfect waveform separation in distributed MIMO radar. IEEE Trans. Signal Process..

[30] Lee H., Kim B.H., Park J.K., Yook J.G. (2019). A novel vital-sign sensing algorithm for multiple subjects based on 24-GHz FMCW Doppler radar. Remote Sens..

[31] Malešević N., Petrović V., Belić M., Antfolk C., Mihajlović V., Janković M. (2020). Contactless real-time heartbeat detection via 24 GHz continuous-wave Doppler radar using artificial neural networks. Sensors.

[32] Rahman M.M., Mdrafi R., Gurbuz A.C., Malaia E., Crawford C., Griffin D., Gurbuz S.Z. Word-level sign language recognition using linguistic adaptation of 77 GHz FMCW radar data. Proceedings of the 2021 IEEE Radar Conference (RadarConf21).

[33] Jin F., Sengupta A., Cao S., Wu Y.J. MmWave radar point cloud segmentation using GMM in multimodal traffic monitoring. Proceedings of the 2020 IEEE International Radar Conference (RADAR).

[34] Sengupta A., Jin F., Zhang R., Cao S. (2020). Mm-pose: Real-time human skeletal posture estimation using mmWave radars and CNNs. IEEE Sens. J..

[35] Chen Z., Li G., Fioranelli F., Griffiths H. (2018). Personnel recognition and gait classification based on multistatic micro-Doppler signatures using deep convolutional neural networks. IEEE Geosci. Remote Sens. Lett..

[36] Ledergerber A., D’Andrea R. (2020). A multi-static radar network with ultra-wideband radio-equipped devices. Sensors.

[37] Avazov N., Hicheri R., Muaaz M., Sanfilippo F., Pätzold M. (2021). A trajectory-driven 3D non-stationary mm-wave MIMO channel model for a single moving point scatterer. IEEE Access.

[38] Ahmad A., Roh J.C., Wang D., Dubey A. Vital signs monitoring of multiple people using a FMCW millimeter-wave sensor. Proceedings of the 2018 IEEE Radar Conference (RadarConf18).

[39] Hicheri R., Avazov N., Muaaz M., Pätzold M. The transfer function of non-stationary indoor channels and its relationship to system functions of LFMCW radars. Proceedings of the 22nd IEEE International Workshop on Signal Processing Advances in Wireless Communications (SPAWC’21).

[40] Ting J.W., Oloumi D., Rambabu K. (2018). FMCW SAR system for near-distance imaging applications - practical considerations and calibrations. IEEE Trans. Microw. Theory Tech..

[41] Meta A., Hoogeboom P., Ligthart L.P. (2007). Signal processing for FMCW SAR. IEEE Trans. Geosci. Remote Sens..

[42] Avazov N., Hicheri R., Pätzold M. A trajectory-driven SIMO mm-Wave channel model for a moving point scatterer. Proceedings of the 2021 15th European Conference on Antennas and Propagation (EuCAP).

[43] Abdelgawwad A., Borhani A., Pätzold M. (2020). Modelling, analysis, and simulation of the micro-Doppler effect in wideband indoor channels with confirmation through pendulum experiments. Sensors.

